# Supplementary material for: Is the fire even bigger? Burnout in 800 medical and nursing students in a low middle income country
Source: PLoS One. 2024 Aug 29;19(8):e0307309. doi: 10.1371/journal.pone.0307309 (PMC11361430; doi:10.1371/journal.pone.0307309)
Supplement: S1 Table — (DOCX) [file pone.0307309.s001.docx]

**Supplementary File**

**
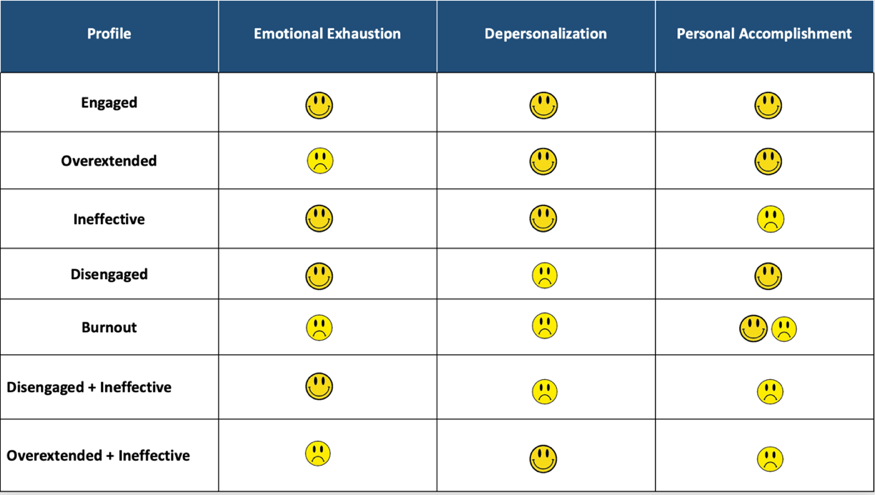
**

**STable1: Various burnout profiles based on subscales of MBI tool.**

All profiles have been deduced from explanation provided by authors of MBI tools [21]
**Key:** = Desired outcome (Low EE/ Low DP/ High PA) ; = Un-desired outcome (High EE/ High DP/ Low PA)
**Abbreviation:** **EE**, Emotional Exhaustion; **DP**, Depersonalization; **PA**, Personal accomplishment
