# Supplementary material for: Is the fire even bigger? Burnout in 800 medical and nursing students in a low middle income country
Source: PLoS One. 2024 Aug 29;19(8):e0307309. doi: 10.1371/journal.pone.0307309 (PMC11361430; doi:10.1371/journal.pone.0307309)
Supplement: S2 Table — (DOCX) [file pone.0307309.s002.docx]

| Variable n (%) | Burnout | | Crude OR | p-value | Adjusted OR | p-value |
| --- | --- | --- | --- | --- | --- | --- |
|  | Yes (n=64) | No (n=315) |  |  |  |  |
| Gender | | | | | | |
| Male (n=186) | 33 (17.7) | 153 (82.3) | 1 | 0.66 |  |  |
| Female (n=193) | 31 (16.1) | 162 (83.9) | 0.89 (0.52, 1.52) |  |  |  |
| Age | 22.17 ±1.62 | 21.47 ± 1.70 | 0.79 (0.67, 0.92) | **0.003** | 0.98 (0.77, 1.25) | 0.87 |
| Year of training | | | | | | |
| Year 1 (n=77) | 5 (6.5) | 72 (93.5) | 1 | **0.001** | 1 |  |
| Year 2 (n=74) | 6 (8.1) | 68 (91.9) | 0.74 (0.35, 1.53) | 0.44 | 0.70 (0.32, 1.52) | 0.136 |
| Year 3 (n=84) | 17 (20.2) | 67 (79.8) | 1.14 (0.53, 2.42) | 0.742 | 1.03 (0.44, 2.41) | 0.95 |
| Year 4 (n=68) | 19 (27.9) | 49 (72.1) | 3.27 (1.21, 8.82) | 0.02 | 2.85 (0.93, 9.83) | 0.076 |
| Year 5 (n=76) | 17 (22.4) | 59 (77.6) | 4.15 (1.45, 11.92) | 0.008 | 3.63 (1.03, 13.31) | **0.048** |
| Smoker | | | | | | |
| No (n=329) | 56 (17.0) | 273 (83.0) | 1 | 0.84 |  |  |
| Yes (n=27) | 5 (18.5) | 22 (81.5) | 1.11 (0.40, 3.05) |  |  |  |
| Drug use | | | | | | |
| No (n=342) | 54 (15.8) | 288 (84.2) | 1 | 0.128 | 1 |  |
| Occasional (n=25) | 8 (32.0) | 17 (68.0) | 2.51 (1.03, 6.11) | 0.043 | 2.36 (1.07, 5.97) | 0.041 |
| Frequent (n=12) | 2 (16.7) | 10 (83.3) | 2.35 (0.42, 11.34) | 0.33 | 2.63 (0.44, 15.91) | 0.29 |
| Accommodation | | | | | | |
| Home (n=179) | 25 (14.0) | 154 (86.0) | 1 | **0.15** | 1 | **0.16** |
| Hostel (n=200) | 39 (19.5) | 161 (80.5) | 1.49 (0.86, 2.58) |  | 1.52 (0.85, 2.68) |  |
| eTable 2: Results of univariate and multivariable analysis of predictors of burnout among medical students. | | | | | | |
