# Supplementary material for: Is the fire even bigger? Burnout in 800 medical and nursing students in a low middle income country
Source: PLoS One. 2024 Aug 29;19(8):e0307309. doi: 10.1371/journal.pone.0307309 (PMC11361430; doi:10.1371/journal.pone.0307309)
Supplement: S3 Table — (DOCX) [file pone.0307309.s003.docx]

| Variable n (%) | Burnout | | Crude OR | p-value | Adjusted OR | p-value |
| --- | --- | --- | --- | --- | --- | --- |
|  | Yes (n=26) | No (n=362) |  |  |  |  |
| Gender | | | | | | |
| Male (n=40) | 1 (2.5) | 39 (91.5) | 1 | **0.285** | 1 | 0.24 |
| Female (n=348) | 25 (7.2) | 323 (92.8) | 3.02 (0.39, 22.90) |  | 3.39 (0.44, 25.80) |  |
| Age | 21.08 ±1.57 | 21.01 ± 1.97 | 0.98 (0.79, 1.21) | **0.86** |  |  |
| Year of training | | | | | | |
| Year 1 (n=134) | 9 (6.7) | 125 (93.3) | 1 | **0.88** |  |  |
| Year 2 (n=111) | 9 (8.1) | 102 (91.9) | 0.87 (0.26, 2.93) | 0.82 |  |  |
| Year 3 (n=75) | 4 (5.3) | 71 (94.7) | 0.71 (0.21, 2.40) | 0.58 |  |  |
| Year 4 (n=68) | 4 (5.9) | 64 (94.1) | 1.11 (0.27, 4.62) | 0.89 |  |  |
| Smoker | | | | | | |
| No (n=361) | 24 (6.6) | 337 (93.4) | 1 | 0.99 |  |  |
| Yes (n=15) | 1 (6.7) | 14 (93.3) | 1.00 (0.13, 7.95) |  |  |  |
| Drug use | | | | | | |
| No (n=379) | 25 (6.6) | 354 (93.4) | 1 | **0.74** |  |  |
| Occasional (n=2) | 0 (0) | 2 (100.0) | 1 (0.99, 1.00) | 0.99 |  |  |
| Frequent (n=7) | 1 (14.3) | 6 (85.7) | 1 (0.99, 1.00) | 0.99 |  |  |
| Accommodation | | | | | | |
| Home (n=121) | 5 (4.1) | 116 (95.9) | 1 | **0.18** | 1 | **0.07** |
| Hostel (n=267) | 21 (7.9) | 246 (92.1) | 1.98 (0.73, 5.38) |  | 2.11 (1.87, 5.84) |  |
| S Table 3: Results of univariate and multivariable analysis of predictors of burnout among nursing students. | | | | | | |
