# Supplementary material for: Is the fire even bigger? Burnout in 800 medical and nursing students in a low middle income country
Source: PLoS One. 2024 Aug 29;19(8):e0307309. doi: 10.1371/journal.pone.0307309 (PMC11361430; doi:10.1371/journal.pone.0307309)
Supplement: Attachment — Submitted filename: PlosOne_Review.docx [file pone.0307309.s004.docx]

Thank you for the opportunity to review this study which is well written. I am sending few suggestions, with the intention of improving the study

1-The final paragraph of the inclusion criteria needs to be revised. It isn´t clear.

2-The authors did not included the limitations of the study

3-References must be reviewed because the journals in which it were published are not described and the authors must try replacement the old references.
