# Supplementary material for: Is the fire even bigger? Burnout in 800 medical and nursing students in a low middle income country
Source: PLoS One. 2024 Aug 29;19(8):e0307309. doi: 10.1371/journal.pone.0307309 (PMC11361430; doi:10.1371/journal.pone.0307309)
Supplement: Attachment — Submitted filename: Response to reviewers.docx [file pone.0307309.s005.docx]

| **Reviewer comment** | **Author response** | **Changes in manuscript** |
| --- | --- | --- |
| **Reviewer #1** | | |
| Clarity and Organization: The overall structure of the discussion is poor-organized, with a not clear introduction, body, and conclusion. However, consider providing a more explicit roadmap at the beginning to guide readers through the main points. | Thank you for your feedback. We have restructured the entire manuscript for enhanced clarity and coherence, including a clearer introduction, body, and conclusion. An explicit roadmap has now been incorporated to better guide readers through the key points. Additionally, we have integrated further discussion points as per the reviewer's suggestions. | Throughout manuscript |
| Depth of Analysis: The analysis of burnout among students is poor, but there's room to delve deeper into the specific factors contributing to burnout. Providing more detailed examples or case studies could enhance the depth of the discussion. | Thank you for your insightful comment. We acknowledge the need for a more in-depth analysis of burnout among students. It's important to note that our study's cross-sectional design and the use of anonymous data preclude the inclusion of individual case studies or detailed examples. Nevertheless, we have included all recorded demographic variables as contributing factors to burnout in our analysis. Additionally, we have expanded the literature review in our discussion to provide a broader context and further elucidate the factors contributing to burnout within the scope of our study's design. | Relevant changes can be found in the methods section and discussion section. |
| Literature Review: While the paper touches on poor literature about student burnout, a more thorough literature review could strengthen the theoretical foundation. Consider incorporating recent studies or alternative perspectives to provide a comprehensive overview of the topic. | Thank you for your feedback. In response, we have carefully expanded the literature review to include recent studies and alternative perspectives, providing a more complete overview of student burnout in the discussion section. | Discussion |
| Methodology and Data: If applicable, discuss the methodology used to gather information on student burnout. Providing details about the research methods or surveys employed will strengthen the credibility of the findings. analysis not done appropriate and suggest to apply new test | Thank you for your comment. We have added more detail about the Maslach Burnout Inventory (MBI) used to investigate burnout among students in the methodology section. We have also included the Cronbach coefficients of the dimensions measured by the MBI tool to the South Asian population to support the credible use of this survey for out study population. We have detailed how the study outcome measures were defined and how the statistical methods were applied based on the guidelines provided by the authors of the MBI tool in the methodology section. | Methods |
| Causation vs. Correlation: Be cautious about attributing causation to certain factors contributing to student burnout. Clearly differentiate between correlation and causation, and acknowledge the complexity of the issue. | Thank you for your insightful comment. We have revised the results and discussion section to reiterate that the factors identified are only associated with burnout and not causative. | Discussion |
| Recommendations: The discussion on potential solutions or recommendations for addressing student burnout is poor. However, consider expanding on the feasibility and practicality of these recommendations. How easily can they be implemented, and what potential challenges might arise? | Thank you for your interesting comment. Implementation of the suggested changes would indeed be met with challenges and it is important for readers to be aware. We have added a small subsection to the discussion highlighting some anticipated challenges. | Discussion section |
| Diversity of Perspectives: It would be beneficial to include diverse perspectives, such as different cultural or demographic influences on student burnout. This will provide a more holistic understanding of the issue. | Thank you for your comment. We have added a few lines to the discussion section identifying and discussing cultural and demographic influences on student burnout in the context of previously published literature. | Discussion Section |
| Future Research: Suggest avenues for future research in the field of student burnout. Identifying gaps in the current understanding and proposing areas for further investigation will contribute to the academic discourse. | Thank you for your comment. We have added a section to the discussion touching about future directions for research on student burnout. | Discussion Section |
| Formatting and Citations: Ensure consistent formatting and citation style throughout the paper. Check for any missing or incorrectly formatted citations to maintain scholarly integrity. | Thank you for your comment. The citations have been revised to maintain uniform formatting throughout the manuscript. | Discussion Section |
| Conclusion: The conclusion effectively poor summarizes the key points. However, consider reiterating the significance of the findings and their implications for educators, policymakers, and other stakeholders. | Thank you for your comment. The conclusion has been revised. | Conclusion Section |
| **Reviewer #2** | | |
| 1- References must be reviewed because the periodical in which it was published does not appear | Thank you for your comment. All references have been double checked, accessed and formatting revised. | References Section |
| 2-The final paragraph of the inclusion criteria should be revised, it is not clear | Thank you for your comment. The eligibility criteria has been revised to include a separate inclusion and exclusion criteria which have been revised for clarity. | Methods Section |
| 3-the authors did not conclude the limitations of the study | Thank you for your comment. A limitations section has been added. | Discussion Section |
| **Reviewer # 3** | | |
| METHODS Medical and nursing students are distinct populations in terms of sex distribution, academic ability (presumably, based on acceptance criteria in other parts of the world), academic stress exposure, duration of study etc. Seeing the sample sizes for both populations were sufficient, it may be better to analyze separately. | Thank you for your insightful comment. The analyses, results and figures have been revised to conduct and report independent findings for medical and nursing students. | Methods, Results and Discussion |
| RESULTS For the survey characteristics and response rate, it may be better to simple report the figures and refrain from making statements of relative size (e.g. 93.8 is greater that 91.7%) if no statistical test has been done to show that these differences are not due to chance. Please reports results for both populations e.g. sex distribution, age etc., rather than lumping them together. | Thank you for your excellent suggestion. The results section has been revised to report findings separately for medical and nursing students. | Results section |
| Authors assert an association between year of study and burnout. Earlier, they reported excluding first year students. The wording in table 3 (year 1 -5) is conflicting. Also, it if nursing students study for fewer years, this is another reason to separate the populations because higher burnout in year 4 for a medical student may have different causes and meaning that the same for a nursing student | Thank you for highlighting this. First year students beginning their training from December 2019 were excluded (clarified in exclusion criteria). Instead, the survey was sent to first years who transitioned to second year during the study duration (January 2020). This confusion was created because final year students graduated in January 2020, while a new class started training in 2019. Rest assured, we included year 1 – year 5 for medical students, and year 1 – year 4 for nursing students. Since this was creating a confusion in the manuscript, we have excluded this. We have separated the analysis for medical and nursing students throughout, including assessment of risk factors in the supplementary analysis. Unfortunately, we chose not to do this for the primary analysis because of lack of power to conduct a meaningful analysis particularly for nursing students. (See supplement Figure 2). | Methods Section |
| **Reviewer #4** | | |
| The study "Is the Fire Even Bigger? Burnout in 800 Medical and Nursing Students in a Low Middle Income Country" is very interesting because it deals with a disorder that is often treated too simply, without taking into account its three-dimensional nature and the multiple variables associated with its development. Although burnout syndrome is an occupational pathology, studies have been described in other settings (volunteers, students, etc.). It should be noted that the process by which a person reaches the most dramatic extreme of this syndrome usually involves a period of chronic exposure to stressors (considered to be an average of around 8 years), so it should be pointed out in the article that the student environment is not the most appropriate. However, the authors highlight statistically significant differences between first year students and fourth year students. | Thank you for the thoughtful and intriguing comment. The authors wish to clarify that the survey implemented was specifically the “Students Survey,” rather than the “Medical Personnel Survey,” adapting a 16-point format to more accurately reflect the student experience rather than the 21-point format used for medical personnel. Although burnout traditionally develops over several years, instances of burnout have been documented in medical and nursing students, as well as resident physicians including interns, who have less than 8 years of exposure to the stressors of the field. The authors agree on the importance of conveying this distinction and have adjusted the article to include that 4-5 years of medical or nursing education may not fully encapsulate the extent of burnout seen in long-term healthcare professionals. These clarifications have been incorporated into the discussion section. | Discussion section |
| It is very interesting that different burnout profiles are described, and I would recommend explaining them “a little better” in the light of the existing literature. Both in the initial summary and in the discussion, authors talk about the distinctions that may arise when studying the phenomenon in "a lower-middle-income country". It is suggested that this aspect be explained in more detail, especially in the discussion section. | Thank you for your comment. We have added a few lines elaborating on what each dimension means in the study outcome measures section. We have also expanded on the literature review in the discussion section to included findings of studies which have reported the burnout dimensions. We have also added a few lines in the discussion section discussing burnout and associated factors particularly in the context of a “lower-middle-income country”. We have also added a small section which discusses anticipated challenged in the implementation of improvement strategies in the context of a lower-middle-income country. | Discussion Section |
| In the Introduction section, authors talk: "previous studies have reported different prevalence rates and gender differences in burnout between medical and nursing students", it is recommended that these differences be explained in a little more detail. It is very wise to follow the recommendations of the authors of the MBI about not using cut-off points, given the great variability by country, profession, age, etc. | Thank you for your comment. We have added to the discussion section to included relevant literature. | Discussion |
| The Discussion section should be more illustrative. There is no need to repeat data that have already been cited in the introduction and in the results section. Simply to interpret in the light of the results obtained in this interesting study. Previous studies on gender, socio-demographic variables, personality types, year of study, student loans, etc. can be also discussed here. | Thank you for your interesting comment. The discussion section has been revised. We have added several more discussion points including comparison to previous literature, proposed reasons as to why we are seeing these differences, burnout in the context of a lower middle-income country, length of exposure, factors associated with burnout and strategies to reduce burnout and anticipated challenges and future direction. We have made efforts to not repeat results unless referring to a comparison from previous literature. | Discussion |
| It is pointed out as a limitation the fact that this is a study carried out in a single centre and it would be advisable to offer data from similar centres in the country, including comparisons between countries. From the dates given, the study was conducted immediately prior to the global COVID-19 pandemic. We imagine that this data could be cross-checked with later data, given the increase in psychological pathologies resulting from the pandemic. | Thank you for your insightful comment. It would be very interesting to compare findings to similar centers in the country and across countries and to post-COVID studies. We have included these to the discussion section as future direction. | Discussion Section |
| The fact that the authors have asked how institutions could help reduce burnout is highly commendable. The suggestions they offer are of great interest and it is recommended to develop them a "little" further, especially on "how to decrease competitiveness without abandoning the possibilities of challenge and challenge necessary for nursing and medical students". | Thank you for your comment. This is a very important aspect. We have added a section discussing the challenges anticipated in the implementation of strategies to promote well-being where we specifically discuss decreasing competitive without taking away the challenge. | Discussion |
| **Editor’s comments** | | |
| 1. No sampling technique | The sampling technique section has been revised. No sampling was needed as all nursing and medical students were included. The minimum sample size required to accurately represent the population was calculated in the methods section | Methods Section |
| 2. Statistical analysis poorly done | We have elaborated on the statistical section and the steps involved. | Methods Section |
| 3. The current burnout cut offs as used in the manuscript does not reflect the categorizations of the MBI tool used | Thank you for comment. We defined cut off for each of the three dimensions by using the means and standard deviations calculated from the scores from our student populations as instructed by the authors of the MBI tool. We have revised the study outcome measures sections in the methods to clarify this further. | Methods Section |
| 4. How was overall burnout measured? | This has been clarified in the manuscript | Methods Section |
| 5. How did the authors arrive at the logistic regression since burnout measurement is a continuous variable? | This has been clarified in the manuscript | Methods Section |
| 6. The abstract is poorly formatted and does not meet the journal guidelines. | Thank you for your comment. We have revised the abstract and formatted it according to journal guidelines. | Abstract |
| 7. In-test citation per the journal requirement is Vancouver and not superscript as in the current format. | Thank you for your comment. We have revised in-text citations to the Vancouver format. | References |
| 8. Failure to report the body that granted ethical approval for the conduct of the study. | Thank you for your comment. We have added this. | Methods Section |
| 9. Tables and figures not labelled | Thank you for your comment. We have labelled tables and figures. |  |
| 10. The introduction needs to be rewritten as the current state does not cover the subject fully. | Thank you for your comment. We have revised the introduction section. | Introduction |
| 11. Improve on the grammar | Thank you for your comment. We have revised the manuscript with correction of grammatical mistakes. | Throughout manuscript. |
| 12. The sample size estimation is questionable. You are dealing with two different populations but used a single population formula to estimate the sample size | This has been clarified in the methods sections. Briefly, this cross-sectional study employed a census approach, eliminating the need for traditional sample size calculations. the minimum sample size required was calculate as noted in the methodology section. |  |
| 13. No inclusion criteria and where the exclusion criteria are should be moved to the participants. | Thank you for your comment. We have included clarified the eligibility criteria in the methods section. |  |
| 14. What qualifies for an incomplete survey? 70%, 80%, or less? | An incomplete survey is where one or more component of the 16-point MBI burnout survey is not missing as this precludes proper calculation of dimension scores. Incomplete answers in demographic survey were not considered incomplete. This clarification has been added to the methods section. |  |
| 15. Refer to the journal's data sharing policy | Thank you for your comment. This has been added. |  |
| 16. The discussion can be improved from the current state. | Thank you for your comment. The discussion has been revised. |  |
