# Supplementary material for: Is the fire even bigger? Burnout in 800 medical and nursing students in a low middle income country
Source: PLoS One. 2024 Aug 29;19(8):e0307309. doi: 10.1371/journal.pone.0307309 (PMC11361430; doi:10.1371/journal.pone.0307309)
Supplement: Attachment — Submitted filename: Response to reviewers2.docx [file pone.0307309.s006.docx]

|  | **Reviewer comment** | **Author response** |  |
| --- | --- | --- | --- |
|  | **Editor Comments** | | |
| 1. | The authors should be specific which of the regression analysis which was employed for this study in the methods. | Thank you for your feedback. In response, we have clarified the specific regression analyses used in our study. We employed both bivariate logistic regression to explore individual relationships between demographic variables and burnout, and multivariable logistic regression to identify independent predictors of burnout, using SPSS Statistics (version 29.0). These details have been added to the methods section for clarity. |  |
| 2. | …thereby limiting our understanding of this complex syndrome. Replace ‘our’ with ‘the’ | Thank you for your comment. This has been changed. |  |
| 3. | Depersonalization assesses a detached response toward one's studies, and Personal Accomplishment evaluates feelings of…. Check when to use capital letters | Thank you for your comment. This has been corrected. |  |
| 4. | Individuals are classified as experiencing high levels of burnout if they score high on EE, high on DP, and either high or low on PA, per guidelines outlined by MBI. The MBI classify burnout as experiencing high EE, DP and low PA. The authors should provide a reference supporting their classification of burnout as experiencing high EE, DP and either high or low PA | Thank you for your comment. As you aptly identified, this is per guidelines by the MBI, the authors of the survey. We have referenced the survey in the text following this statement. |  |
| 5. | The authors claim they used the census approach for the recruitment of study participants. hence, sample size calculation was not applicable in their study but still go ahead to provide sample size estimation in their study. An explanation would be very much appreciated. Again, the sample size formula in the work is not appropriate especially when the authors are dealing with two populations and have provided values (p1 and p2) for the two populations | Thank you for your insightful comments. We have revised the manuscript to exclude the sample size calculation section, as a census approach was utilized, making sample size calculation unnecessary. This should clarify our recruitment strategy. |  |
| 6. | There is no need to create a separate subheading for response rate. The whole sentence under the subheading can be merged with the demographic characteristics | Thank you for your comment. This has been changed. |  |
| 7. | How can Institutions help to reduce burn out and promote well-being? Challenges anticipated in implementation of strategies to promote well-being: These subheadings were not assessed in the current study and could mislead readers. This could be capture under study implications. | Thank you for your comment. The heading for challenges was originally added as per a reviewer’s suggestion in the first review. To best consolidate these two comments, the headings have been removed but the content has been retained. |  |
| 8. | The statistical analysis still needs to be clearer as the current state makes replication difficult. | Thank you for your feedback. We have revised the statistical analysis section to provide more detailed and clear descriptions of the methods used, to improve transparency and ease of replication. The revised section now includes specific tests used, the rationale for their selection, and detailed steps of the analysis process. |  |
| 9. | Our findings show that only one-third of the student population was engaged….what does this means? | Thank you for your comment. Results show that only 32.6% of the students felt engaged (Table 2) which is defined by the Maslach burnout inventory as low scores on the emotional exhaustion and depersonalization subscales and high scores on the personal accomplishment subscale. We have added this explanation to the text as well. |  |
| 10. | The discussion can still be improved. Especially, the authors have made some bold statements without providing references and also repeating some of the statements in the introduction in the discussion. The discussion is meant to discuss the study results by comparing with literature and giving explanations where necessary as well giving an implication of the results. | Thank you for your feedback. The discussion appeared a bit scattered. We have revised and simplified this section while doing our best to retain the additions that were requested by reviewers in the previous review cycle. We have organized it as follows:  -Summary of findings  -Significance and consequences of burnout  -Comparison of subscales scores to previous literature  -Comparison of burnout profiles to previous literature with explanation of differences  -Comparison of risk factors associated with burnout in general and specific to the South Asian population to previous literature  -Study implications  -Strengths/limitations and future direction |  |
|  | **Reviewer # 5 Comments** | | |
| Many thanks to adding to the literature. It is now obvious that healthcare workers, regardless of geography, are affected by burnout. It's time to move on finding solutions. The authors have done a great job of rewriting their paper. All of my comments are to improve readability and flow. | | | |
| 1. | 27: Change to ...67.5% (517/727) of medical and nursing students... It changes the emphasis of the sentence onto the results. | Thank you for your comment. This has been changed. |  |
| 2. | 39: Change ...We aim to describe... to ... We describe... You have described after all. | Thank you for your comment. This has been changed. |  |
| 3. | 48: I'm conflicted about the use of the shorthand to describe the parts of burnout. Healthcare workers reading the paper will read PE as Pulmonary Embolism so using it for personal efficacy increases the cognitive load of the reader. | Thank you for your comment. We agree, it is confusing, and PE does cognitively default to pulmonary embolism. For this, we initially contemplated referring to personal efficacy as personal accomplishment and abbreviating as PA.  However, the authors of the MBI tool have described this specifically as personal efficacy and explain that this refers to a student’s satisfaction with their accomplishments rather than accomplishments alone. <https://www.mindgarden.com/313-mbi-general-survey-for-students>  We have now changed PE to PF to fix this. |  |
| 4. | 56: Change ...stidents were engaged... to ...students felt engaged... | Thank you for your comment. This has been changed. |  |
| 5. | 61: Change ...other risk factors... to ...other risk factors for burnout... | Thank you for your comment. This has been changed. |  |
| 6. | 63: Change ...Two-thirds of our participants suffered from at least one burnoiut dimension... to ...Two-thirds of our participants experienced at least one dimension of burnout... | Thank you for your comment. This has been changed. |  |
| 7. | 71: Change ...the three dimensions of burnout... to ...the three burnout dimensions... | Thank you for your comment. This has been changed. |  |
| 8. | 74: Change ...manifesting in a myriad... to ...with many... | Thank you for your comment. This has been changed. |  |
| 9. | 75: What exactly do you mean by metabolic disorders? Diabetes? Cardiovascular disease? | Thank you for your comment. Metabolic disorders are referring to diabetes and metabolic syndrome. This has been added to the text. |  |
| 10. | 83: Reword ...The pressures of competition for higher performance... as it doesn't make sense as a sentence. Perhaps just say ...The pressures of competition... | Thank you for your comment. This has been changed. |  |
| 11. | 85: Change ...the vulnerability of students... to ...a student's vulnerability... | Thank you for your comment. This has been changed. |  |
| 12. | 94: Remove the word ...Furthermore... - it adds nothing to the sentence | Thank you for your comment. This has been changed. |  |
| 13. | 95: Remove ...often... | Thank you for your comment. This has been changed. |  |
| 14. | 96. Split into two sentences ....single-measures. These oversimplify... | Thank you for your comment. This has been changed. |  |
| 15. | 97. Changing ...Using... to ...Using the results of the MBI... | Thank you for your comment. This has been changed. |  |
| 16. | 101. Change ...utilized... for used. Less syllables increases readability | Thank you for your comment. This has been changed. |  |
| 17. | 107. Change ... The aim of our study... to ...Our study aimed... | Thank you for your comment. This has been changed. |  |
| 18. | 115. Remove ...a period of... It's reductive | Thank you for your comment. This has been changed. |  |
| 19. | 122. Replace ...Students... with ...Those... You've already used the word students in the previous sentence | Thank you for your comment. This has been changed. |  |
| 20. | 128. Change ...The e-mail informed... with ...Students were informed... | Thank you for your comment. This has been changed. |  |
| 21. | 130. Change ...had their e-mails removed... to ...students were removed... | Thank you for your comment. This has been changed. |  |
| 22. | 131. ...The survey was incentivized... should be a new paragraph. | Thank you for your comment. This has been changed. |  |
| 23. | 145. Change ...MBI burnout tool... to ... The MBI tool. Remember what MBI stands for. If you write out your sentence it would read Maslach Burnout Inventory Burnout Tool. | Thank you for your comment. This has been changed. |  |
| 24. | 148. Emotional Exhaustion describes feelings of ... it doesn't measure them. | Thank you for your comment. This has been changed. |  |
| 25. | 152-154. This could be clearer. | Thank you for your comment. The sentence have been revised. |  |
| 26. | 160. Incorrect spelling of Categorized | Thank you for your comment. This has been changed. |  |
| 27. | 179-181. Would sound better as ...and ensure findings accurately respresent the populations attribute prevalence, we calculated the minimal sample size using the formula... | Thank you for your comment. The sample size section was deleted per editors comment therefore this change couldn’t be made. |  |
| 28. | 200. Easier to say .. in each group... rather than ... in both groups together and seperately... | Thank you for your comment. This has been changed. |  |
| 29. | 231. Change ...the data cannot be made available publically... to ...is not available publically... | Thank you for your comment. This has been changed. |  |
| 30. | 241. Make a new sentence of ...The average completion time... Though I would suggest removing this as it is irrelevent data that adds nothing to your narrative. You don't suggest it is a statistically significant difference after all | Thank you for your comment. This sentence was deleted. |  |
| 31. | 253. Should be ...The majority were single and 60.9% lived on campus... | Thank you for your comment. This has been changed. |  |
| 32. | 254. What does endorsed mean? Does it mean that said drugs were okay or does it mean they used them? Clearer wording would be ... 3.5% used recreational substances occassioanlly and 2.5% used it more than X times a week... You need to let the reader know what the difference. isbetween occassional and frequent use. | Thank you for your comment. This has been changed. |  |
| 33. | 259. It's clearer if written as ...4% (15/767) students did not dicslose their smoking status... | Thank you for your comment. This has been changed. |  |
| 34. | 263. Again, you use the term endorse when it's meaning is not clear. | Thank you for your comment. This has been changed. |  |
| 35. | 271. Change to ...Based on this cut-off... | Thank you for your comment. This has been done for medical and nursing students. |  |
| 36. | 288-289. It is more powerful to say ...The majority of medical students felt ineffective and burnt out... | Thank you for your comment. This has been changed. |  |
| 37. | 292. Again, the medical students felt engaged rather than were engaged. | Thank you for your comment. This has been changed. |  |
| 38. | 295. Same again. | Thank you for your comment. This has been changed. |  |
| 39. | 304. Change ...compared to... to ...than... | Thank you for your comment. This has been changed. |  |
| 40. | 306. Would be clearer to start a new sentence as ... Occasional drug use also appeared to be a risk factor... | Thank you for your comment. This has been changed. |  |
| 41. | 313. Students ...felt... engaged | Thank you for your comment. This has been changed. |  |
| 42. | 318. Change ...risk factors found to be associated... to ... were associated with... | Thank you for your comment. This has been changed. |  |
| 43. | 336. Change the sentence order to make it more powerful. ...The prevalence of the burnt out profile was lower in our cohort than previously reported... | Thank you for your comment. This has been changed. |  |
| 44. | 346. ...the authors of the MBI advise against this as... | Thank you for your comment. This has been changed. |  |
| 45. | 354. You can make this clearer by saying ... Previous studies have reported a number of factors associated with burnout. These include... | Thank you for your comment. This has been changed. |  |
| 46. | 359. This could be more cleqrly expressed as ...The longer youy are exposed to stressors, the more likely you are to experience burnout... | Thank you for your comment. This has been changed. |  |
| 47. | 363. Use less words ..healthcare professionals readuating... | Thank you for your comment. This has been changed. |  |
| 48. | 369. Spelling of interdependent | Thank you for your comment. This has been changed. |  |
| 49. | 377. Did you capture any data around parental occupation in your cohort? | Thank you for your comment. No, we did not record parental occupation. |  |
| 50. | 379. ...Many... not ...Multiple... | Thank you for your comment. This has been changed. |  |
| 51. | 380. ...While many are inconclusive... | Thank you for your comment. This has been changed. |  |
| 52. | 383. ...Use... not ...Utilization... | Thank you for your comment. This has been changed. |  |
| 53. | 389. You can make this punchier. Try ...Institutions should take a multi-promged approach in targetting student burnout. This includes readily available, anonymous counselling and support services... | Thank you for your comment. This has been changed. |  |
| 54. | 394. Don't hide a new argument in the middle of the paragraph. New point = new para | Thank you for your comment. New paragraph has been started. |  |
| 55. | 405. Think of the international reader. What is unique about your circumstances in Pakistan? What do you mean by 'significant youth bulge'? | Thank you for your question. The authors were referring to a greater proportion of younger people in Pakistan, given lower life expectancy and higher birth rates compared with a developed country. However, this paragraph has now been revised to simply and organize the discussion as suggested by the editor comments and this phrase is now excluded. |  |
| 56. | 423. You want to close strongly. ...Identifying the drivers of burnout unique to LMICs is vital. Future initiatives must include... | Thank you for your comment. This has been changed. |  |
| 57. | 426-427. This is a very soft ending given you state earlier that it is key. Perhaps ...Faculty training on psychologically safe student evaluation and feedback is vital... | Thank you for your comment. This has been changed. |  |
|  | **Reviewer #6 Comments** | | |
| Thank you for your interest in this important research area. This is very relevant as has potential impact on the quality of care which these students will ultimately deliver in the healthcare setup. Your report is clearly presented, however, I have minor observations: | | | |
| 1. | Consider rephrasing lines 70-72; “Therefore, it is imperative to identify and address the ongoing stressors associated…..” | Thank you for your comment. This sentence has been revised. |  |
| 2. | Line 107: If I may ask, why was the survey incentive awarded to only the 5th participant and not all survey participants? | Thank you for your question. This was mainly due to limited funds for the study and the budget did not allow for every participant to receive the study incentive. |  |
